# Comparison of ACDF with stand-alone PEEK cage versus zero-p implant for the treatment of cervical degenerative disease: a systematic review and meta-analysis

**DOI:** 10.3389/fsurg.2026.1945489

**Published:** 2026-09-03

**Authors:** Yiheng Zhao, Juan Xie, Ling Zhu, Jianna Zhang, Junhui Zhang

**Affiliations:** 1 Department of Neurosurgery, West China School of Public Health and West China Fourth Hospital, Sichuan University, Chengdu, China; 2 Department of Emergency Medicine, West China Hospital, Sichuan University/West China School of Nursing, Sichuan University, Chengdu, China; 3 Disaster Medical Center, Sichuan University, Chengdu, China; 4 Nursing Key Laboratory of Sichuan Province, Chengdu, China; 5 Department of Thyroid and Breast Surgery, West China School of Public Health and West China Fourth Hospital, Sichuan University, Chengdu, China

**Keywords:** anterior cervical discectomy and fusion, cervical degenerative disease, meta-analysis, stand-alone peek cage, systematic review, zero-P implant

## Abstract

**Introduction:**

In recent years, stand-alone polyetheretherketone (PEEK) cages (SA) and Zero-P implants (ZP) have been widely adopted clinically in anterior cervical discectomy and fusion (ACDF) for cervical degenerative disease (CDD). However, which is superior between SA and ZP remains highly controversial. This meta-analysis was designed to conduct a head-to-head comparison of the safety, clinical efficacy, and radiological effectiveness of ACDF with ZP and SA.

**Methods:**

This research was carried out in accordance with the PRISMA Statement. Systematic retrieval of eligible literature was completed covering three core electronic bibliographic databases: PubMed, Web of Science Core Collection, and Embase up to February 19, 2026. For dichotomous outcomes, including the fusion rate and subsidence rate, the effect sizes were calculated as the relative risk (RR) with corresponding 95% confidence intervals (CIs). For continuous outcomes, including surgical time, blood loss, disc height, vertebral height, C2-7 Cobb angle, segmental Cobb angle, VAS score, and NDI score, the effect sizes are expressed as the mean difference (MD) with 95% CIs.

**Results:**

Eight eligible studies comparing ACDF with SA and ZP for CDD treatment were ultimately included. The pooled patient cohort consisted of 727 participants in total, with 384 patients assigned to the SA group for ACDF and 343 patients allocated to the ZP group. Compared with the ZP, the SA had a significantly shorter operative time and higher NDI score. The segmental Cobb angle, disc height and vertebral height of the SA were significantly lower than those of the ZP. The SA group had a significantly higher subsidence rate than the ZP group, with no remarkable difference observed in the C2-7 Cobb angle, fusion rate, VAS score between the two cohorts.

**Discussion:**

Current pooled findings suggest that SA may be associated with shorter operative time, while ZP may correlate with lower subsidence rate and better preservation of cervical alignment. As all available evidence is derived exclusively from observational studies and rated as low to very low certainty, the conclusions must be interpreted with caution.

**Clinical Trial Registration:** https://www.crd.york.ac.uk/PROSPERO/view/CRD420261325943, PROSPERO, CRD420261325943.

## 1 Introduction

Since Smith and Robinson (1) first proposed the use of anterior cervical discectomy and fusion (ACDF) for the treatment of cervical degenerative disease (CDD) in 1958, ACDF has long been regarded as the gold standard for treating CDD. Despite more than 60 years having passed, ACDF remains recognized as a classic surgical procedure for CDD. Anterior cervical plates have been widely used in ACDF procedures because of their ability to improve cervical curvature, provide excellent immediate stability, and increase fusion rates (2–4). However, scholars have gradually reported that the use of anterior cervical plates is also associated with a series of complications, including the following postoperative complications: adjacent segment degeneration (ASD), hardware-related adverse events (screw loosening and fracture), prevertebral soft tissue irritation and iatrogenic injury, and postoperative dysphagia (5–8).

To avoid a series of complications caused by anterior cervical plates, the use of anterior cervical plating has gradually been abandoned in clinical practice. In recent years, stand-alone polyetheretherketone (PEEK) cages (SA) and Zero-P implants (ZP) have been widely adopted clinically (9–12). Because they are contained within the intervertebral space and do not protrude beyond the vertebral body surface, they can reduce irritation to the anterior cervical soft tissues and lower the incidence of dysphagia (13, 14). SA can restore intervertebral height, improve cervical lordosis, and facilitate interbody fusion (15, 16). However, some studies have reported risks, including cage migration, subsidence, and the need for reoperation (17). Moreover, owing to the integrated plate and screws placed within the intervertebral space, ZP can improve immediate postoperative stability, with favorable clinical outcomes and satisfactory safety (18). Nevertheless, some studies have reported issues such as a higher subsidence rate than with anterior cervical plating (19) and failure to effectively maintain cervical curvature in the treatment of multilevel cervical spondylosis (20, 21).

Both stand-alone PEEK cages and Zero-P implants are commonly used in current ACDF surgery. However, which is superior between SA and ZP remains highly controversial. Few meta-analyses directly compare the safety and clinical and radiological efficacy of SA vs. ZP for the treatment of CDD. This meta-analysis was designed to conduct a head-to-head comparison of the safety, clinical efficacy, and radiological effectiveness of ACDF using ZP and SA.

## 2 Materials and methods

### 2.1 Study registration and search strategy

This research was performed in accordance with the PRISMA 2020 Statement (22). It was registered on the PROSPERO platform (CRD420261325943). Systematic retrieval of eligible literature was completed covering three core electronic bibliographic databases: PubMed, Web of Science Core Collection, and Embase, covering all relevant studies published from the establishment of each database up to February 19, 2026. The search strategy combined the following index terms for title or abstract screening: (cervical) AND (standalone OR stand-alone OR stand alone) AND (stand-alone anchored spacer OR anchored cage OR self-lock* OR SAAS OR zero-p OR zero-profile OR no-profile OR anchored spacer). The complete search strategies are presented in Supplementary Table S1. No language restrictions were imposed during the search process. To achieve more comprehensive inclusion of eligible literature, PEEK or polyetheretherketone was not adopt in the search strategy. Instead, whether the cage was made of PEEK by was determined by full-text reading.

### 2.2 Inclusion and exclusion criteria

This study included comparative research encompassing randomized controlled trials (RCTs) and nonrandomized cohort studies, which primarily aimed to compare the differences in safety, clinical efficacy and radiological findings between SA and ZP for ACDF in patients with CDD. The exclusion criteria included irrelevant studies, review articles, research focusing on adjacent segment disease, comparative studies of SA or ZP with other devices, studies with overlapping patient populations, noncomparative studies, research involving hybrid surgical approaches, conference abstracts, biomechanical studies, and studies where patients were diagnosed with other diseases.

### 2.3 Data extraction

Relevant data from all included studies were independently extracted by two trained researchers: (1) number of surgeries and bone graft types; (3) age, sex and follow-up period; (4) surgical time and blood loss; (5) disc height, vertebral height, C2-7 Cobb angle and segmental Cobb angle; (6) fusion rate and subsidence rate; and (7) VAS and NDI score.

### 2.4 Risk of bias assessment

Two reviewers independently applied the Cochrane ROBINS-I tool (23) to evaluate the bias risk of all enrolled non-randomized cohort studies across seven domains: confounding, selection of participants, classification of interventions, deviations from intended interventions, missing data, outcome measurement, and selective reporting. Each domain was categorized into four risk grades: low, moderate, serious, and critical. Any disagreements between the two assessors were resolved by iterative discussion with a third senior author.

### 2.5 Statistical analysis

All statistical analyses for the present systematic review and meta-analysis were undertaken using the Review Manager software (RevMan version 5.3; Cochrane Collaboration, London, UK). The absolute postoperative values of C2-7 Cobb angle, segmental Cobb angle, vertebral height, disc height, VAS score and NDI score recorded at the final follow-up timepoints were extracted from each included study. For dichotomous outcomes, including the fusion rate and subsidence rate, the effect sizes were calculated as the relative risk (RR) with corresponding 95% confidence intervals (CIs). For continuous outcomes, including surgical time, blood loss, disc height, vertebral height, C2-7 Cobb angle, segmental Cobb angle, VAS score, and NDI score, the effect sizes are expressed as the mean difference (MD) with 95% CIs. For all statistical tests in this meta-analysis, the threshold of statistical significance was set at a two-tailed *P* value below 0.05. Meanwhile, between-study heterogeneity among the enrolled literature was quantified and evaluated using the I^2^ statistic (which ranged from 0 to 100%), and an I^2^ value >50% indicated significant heterogeneity. For all pooled analyses in this meta-analysis, a random-effects model was adopted in the presence of statistically significant between-study heterogeneity; by contrast, a fixed-effects model was selected for data merging when the I^2^ statistic was less than 50%. Funnel plot was generated to examine the risk of publication bias across included studies when the number of included studies exceeded 10, and additionally performed sequential sensitivity analysis to assess the stability and reliability of our overall pooled outcomes. Meta-regression was conducted to test the stability of the results when the number of enrolled studies exceeded 10. Subgroup analysis was conducted according to the number of surgical levels and number of screws in ZP. Two independent investigators conducted the evidence certainty assessment pursuant to the Grading of Recommendations, Assessment, Development, and Evaluation (GRADE) framework (24).

## 3 Results

### 3.1 Identification of relevant studies

**Figure 1:**
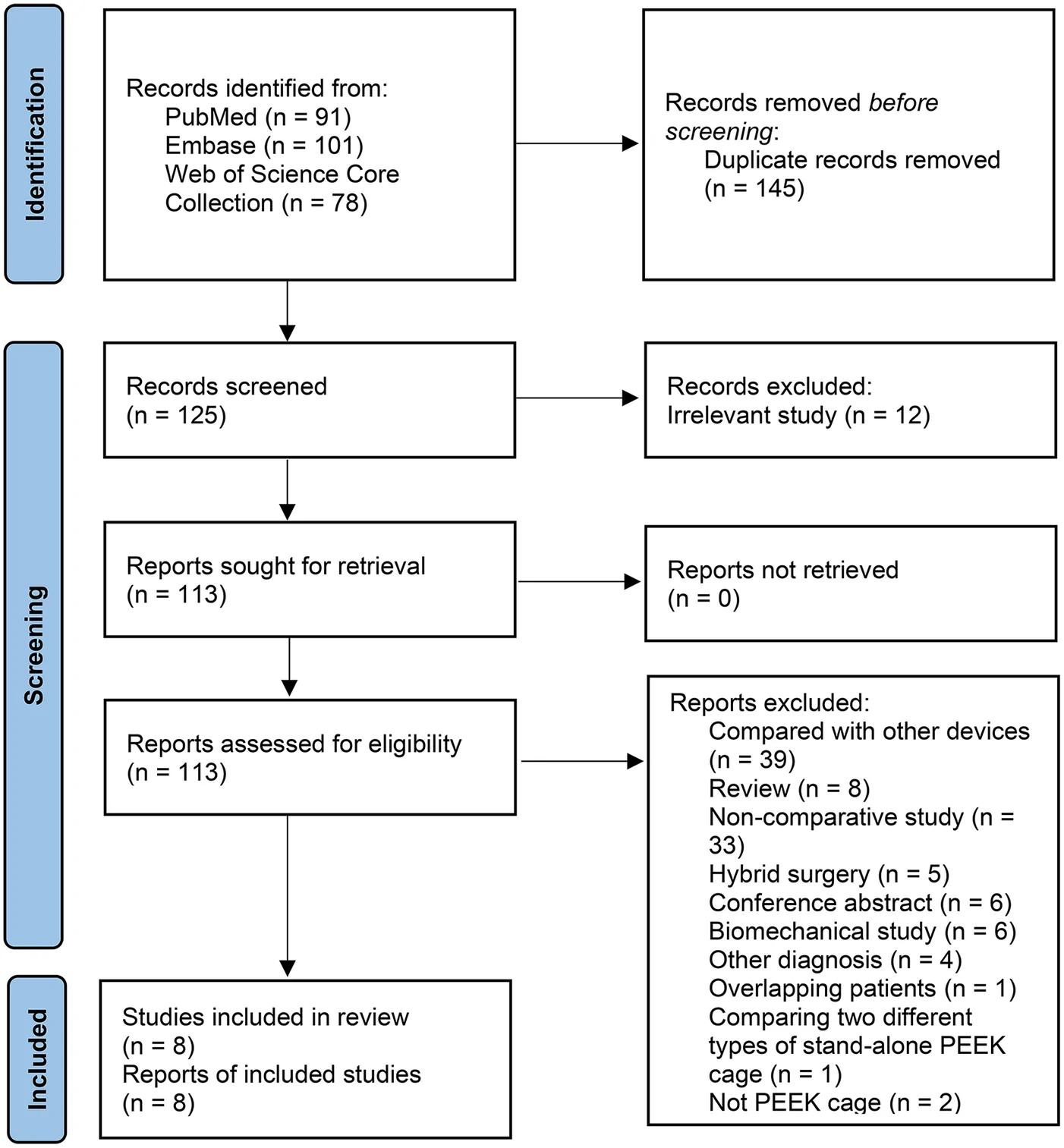
A flow diagram of study selection.

A total of 270 studies were initially retrieved through systematic searches of the PubMed, Web of Science core collection and Embase databases. After duplicate records were removed, 125 studies remained for further screening. We subsequently excluded 12 irrelevant studies, 8 review articles, 39 comparative studies of SA or ZP with other surgical devices, 33 noncomparative studies, 5 studies involving hybrid surgical approaches, 6 conference abstracts, 6 biomechanical studies, and 4 studies enrolling patients with other concurrent diseases. One study with potential overlapping patient cohorts was excluded (25), along with 1 study that compared two different types of SA (26). Meanwhile, after re-examining the included studies, we found that Mu et al. (27) and Virkar et al. (28) did not explicitly report cage material. Although their radiographic images suggested PEEK cages, after careful consideration, we could not rule out the possibility that titanium cages might have been included. Consequently, these two studies were excluded. Finally, 8 eligible studies comparing ACDF with SA vs. ZP for the treatment of CDD were included in this study (29–36). The PEEK cage was used across all included studies. The detailed study selection process is presented in Figure 1.

### 3.2 Characteristics of the included studies

All the studies ultimately eligible for this meta-analysis were observational cohort studies, including 7 retrospective analyses and 1 prospective cohort investigations. The core baseline features of the included studies, including study design, material of cage, follow-up duration, patient demographic data and surgical intervention details, are systematically summarized in Table 1. The pooled patient cohort consisted of 727 participants in total, with 384 patients assigned to the SA group for ACDF and 343 patients allocated to the ZP group.

**Table 1:** Basic information of the included studies.

Study	Study design	Country	Surgical level	Material of cage	Screw number in ZP	Sample size		Mean age (y)		Gender (M/F)		Follow-up (month)	
						SA	ZP	SA	ZP	SA	ZP	SA	ZP
Balakumar (29)	RCS	UK	1/2/3	PEEK	2-screw	83	79	53.8 ± 10.07	51.16 ± 12.12	38/45	47/32	9.5 ± 5.8	9.08 ± 6.6
Cho (30)	RCS	Korea	1	PEEK	4-screw	29	21	55.2 ± 10.4	56.1 ± 12.0	19/10	12/9	24	
Gerszten (31)	RCS	USA	3/4	PEEK	3-screw	35	33	52 (42–70)	60 (41–75)	15/8	16/7	>6	
Khattab (32)	PCS	Egypt	1	PEEK	2-screw	21	29	53.2 ± 5.4	54.4 ± 5.3	9/12	10/19	24	
Kwon (33)	RCS	Korea	1	PEEK	4-screw	20	21	52.20 ± 10.37	49.55 ± 9.34	10/10	9/12	12	
Lee (34)	RCS	Korea	1	PEEK	4-screw	60	23	53.62 ± 11.47	57.26 ± 13.28	30/30	11/12	18.98 ± 10.30	12.57 ± 2.09
Mckee (35)	RCS	UK	1	PEEK	2-screw	116	117	45.0 ± 7.71	46.2 ± 7.79	55/61	58/59	7.2	8.2
Shin (36)	RCS	Korea	1	PEEK	4-screw	20	20	49.0 ± 11.0	50.0 ± 12.0	12/8	7/13	13.1 ± 1.2	13.2 ± 1.0

RCS, retrospective cohort study; PCS, prospective cohort study; SA, stand-alone; PEEK cage; ZP Zero-P implant; DBM, demineralized bone matrix; NS, not specified.

### 3.3 Quality assessment

**Figure 2:**
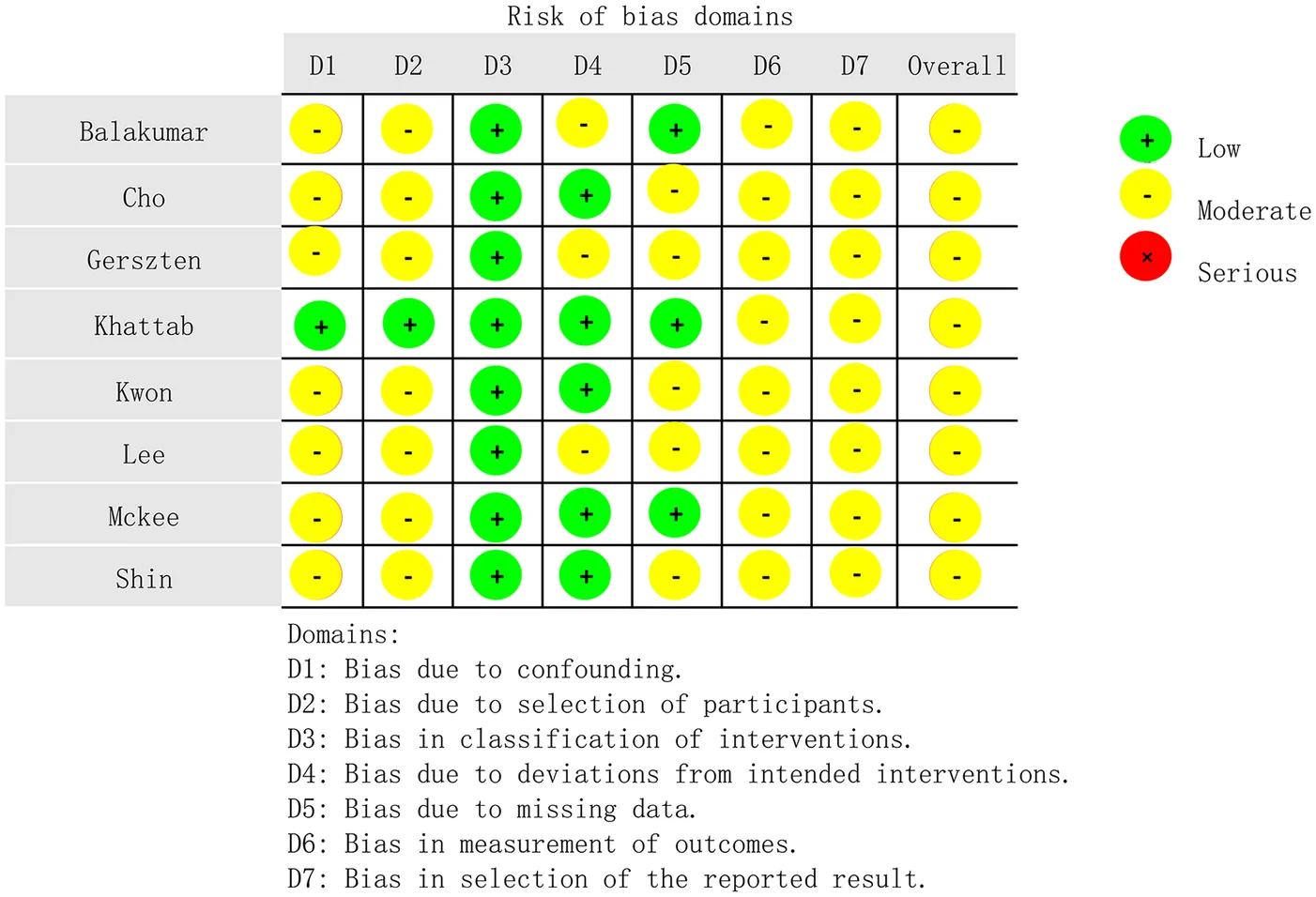
Risk of bias assessment of included cohort studies using the ROBINS-I tool.

Methodological bias of the 8 cohort studies was appraised via the ROBINS-I instrument. All cohort studies were determined to be at moderate risk of bias. This suggests that these studies presented partial methodological deficiencies, and their conclusions warrant cautious interpretation. The full results of the methodological quality assessment are displayed in Figure 2.

### 3.4 Meta-analysis of outcomes

#### 3.4.1 Surgical outcomes

**Figure 3:**
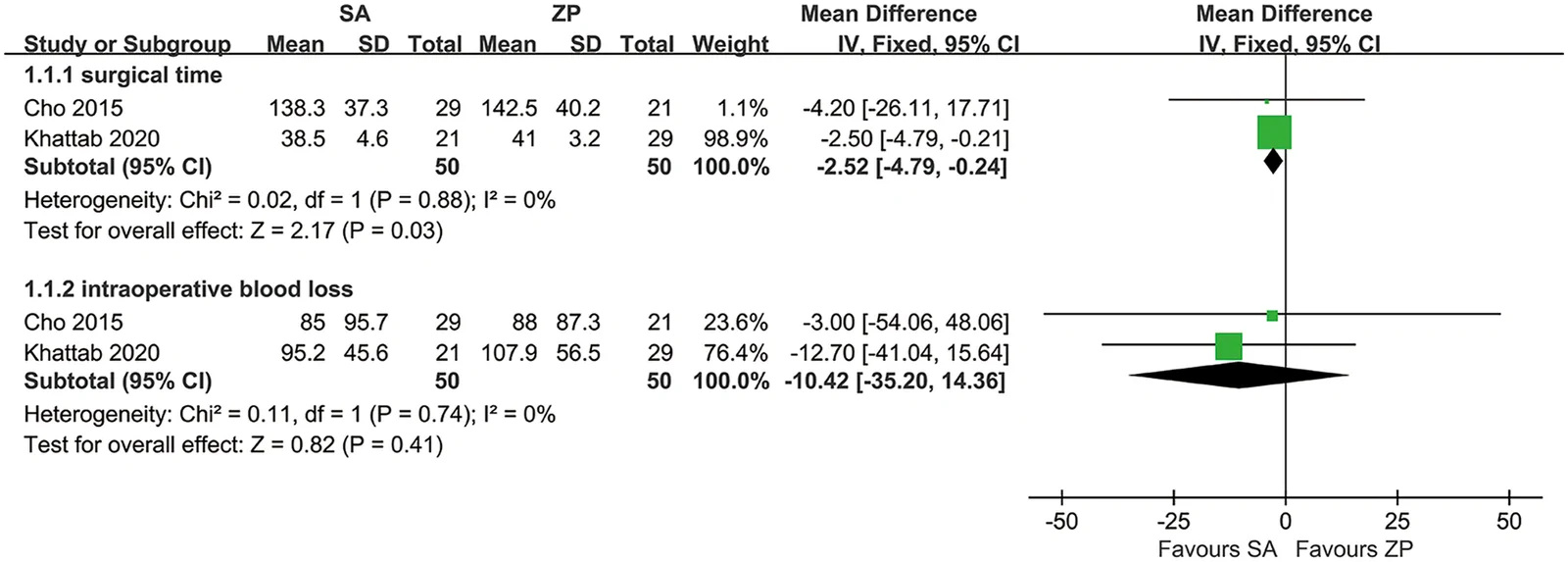
Comparison of surgical outcomes.

Compared with the ZP group, the SA group had a significantly shorter operative time (MD −2.52, 95%CI −4.79 to −0.24; *P* = 0.03; I^2^ = 0%). No significant difference was identified in intraoperative blood loss (MD −10.42, 95%CI −35.20 to −14.36; *P* = 0.41; I^2^ = 0%) (Figure 3).

#### 3.4.2 C2-7 cobb angle, segmental cobb angle, disc height and vertebral height

**Figure 4:**
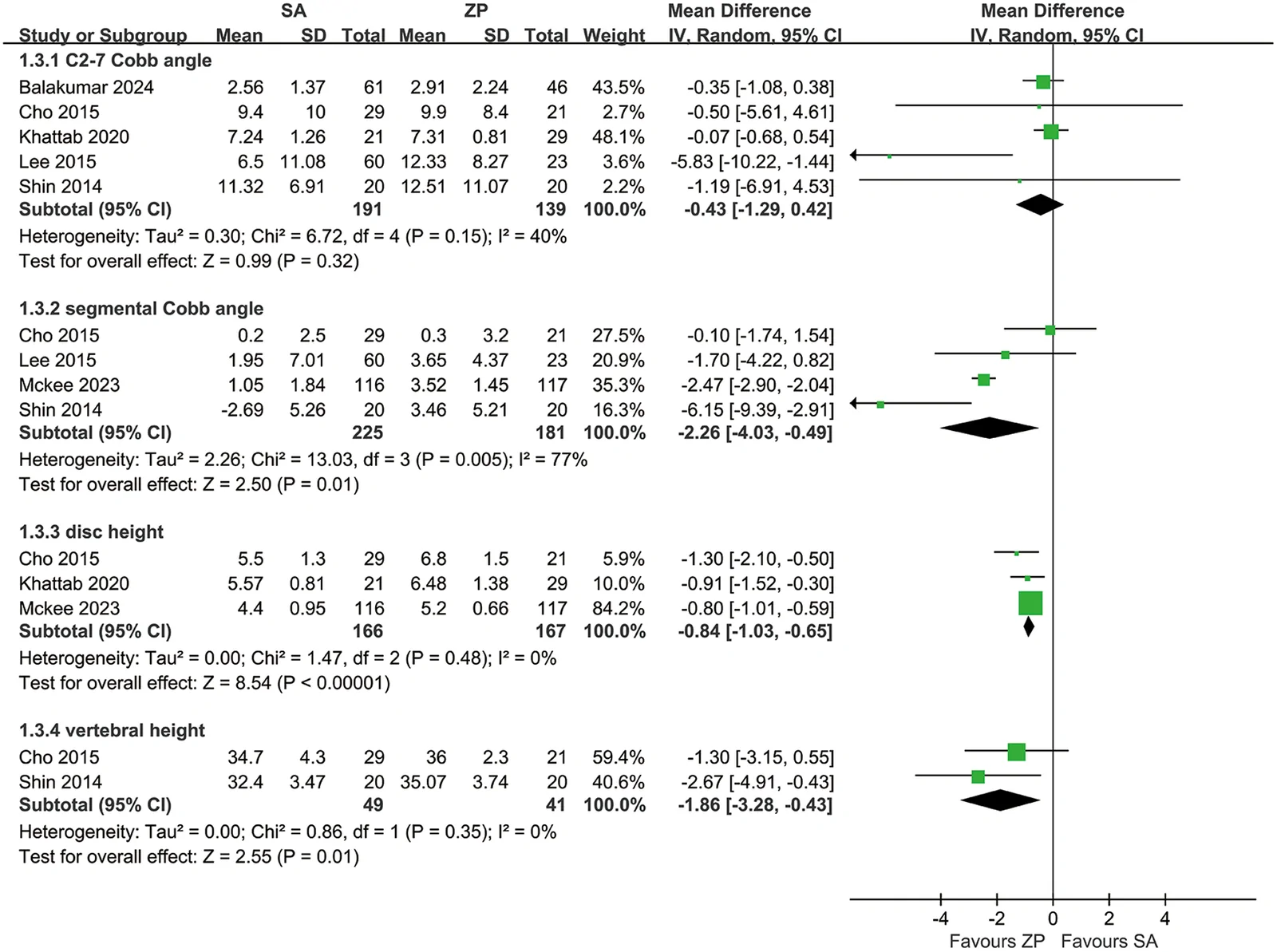
Comparison of cervical alignment.

Pooled analysis of radiographic outcomes revealed no significant difference in the C2-7 Cobb angle between the SA cage group and the ZP group (MD: −0.43, 95% CI [−1.29, 0.42], *P* = 0.32; I^2^ = 40%; Figure 4). In contrast, the segmental Cobb angle of the SA was significantly smaller than ZP (MD: −2.26, 95% CI [−4.03, −0.49], *P* = 0.01; I^2^ = 77%; Figure 4). Additionally, compared with patients who received ZP, patients who received SA had significantly shorter disc heights (MD: −0.84, 95% CI [−1.03, −0.65], *P* < 0.00001; I^2^ = 0%; Figure 4) and vertebral heights (MD: −1.86, 95% CI [−3.28, −0.43]; *P* = 0.01; I^2^ = 0%; Figure 4).

**Figure 5:**
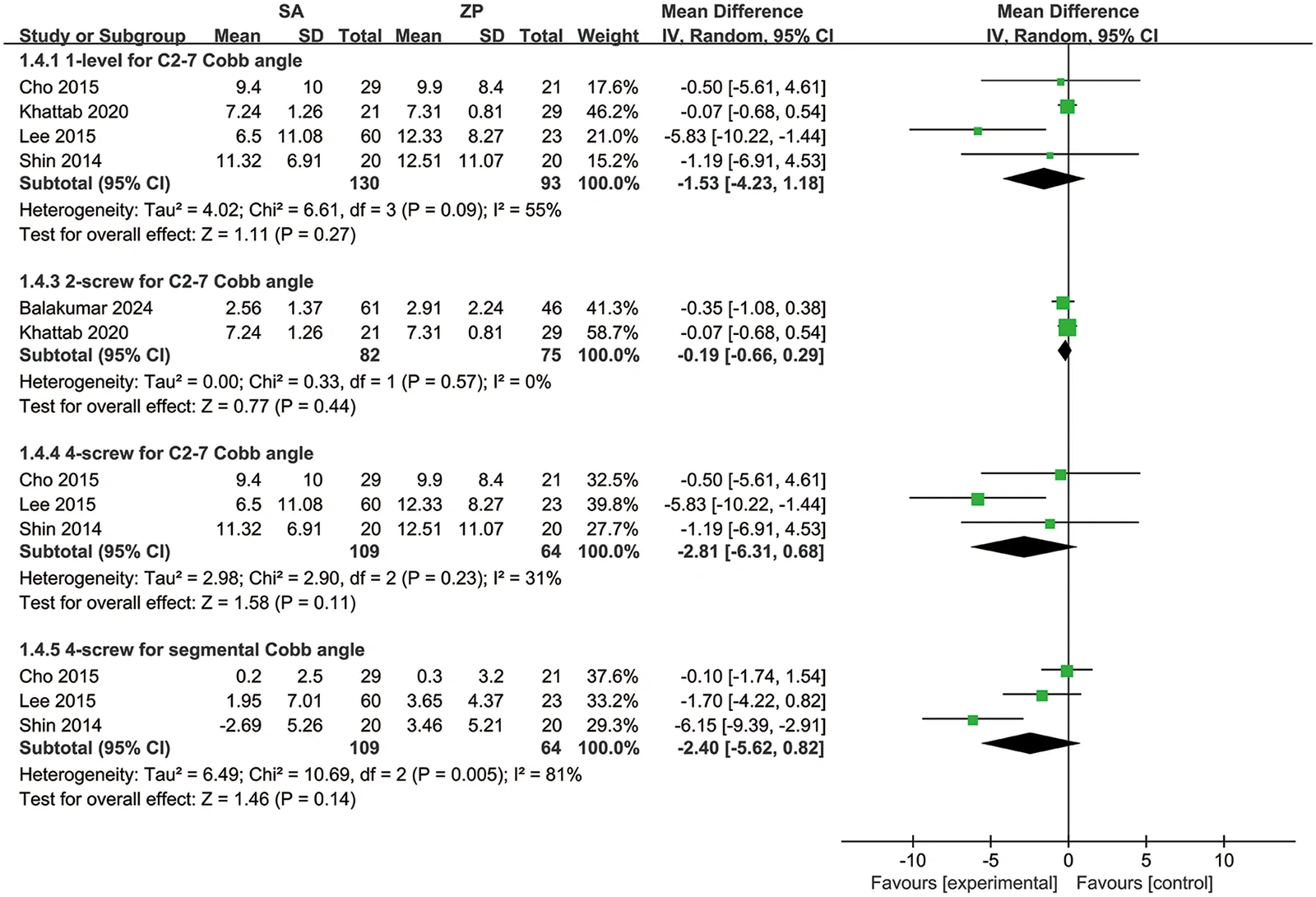
Subgroup analysis of C2-7 Cobb angle and segmental cobb angle according to number of surgical level and screws in ZP.

No statistical difference was identified in C2-7 Cobb angle between the two groups in subgroup analysis of 1-level surgery (MD −1.53, 95%CI −4.23 to 1.18; *P* = 0.27; I^2^ = 55%), 2-screw ZP (MD −0.19, 95%CI −0.66 to 0.29; *P* = 0.44; I^2^ = 0%), 4-screw ZP (MD −2.81, 95%CI −6.31 to 0.69; *P* = 0.11; I^2^ = 31%; Figure 5).

No statistical difference was identified in segmental Cobb angle between the two groups in subgroup analysis of 4-screw ZP (MD −2.40, 95%CI −5.62 to 0.82; *P* = 0.14; I^2^ = 81%; Figure 5).

#### 3.4.3 Clinical outcomes

**Figure 6:**
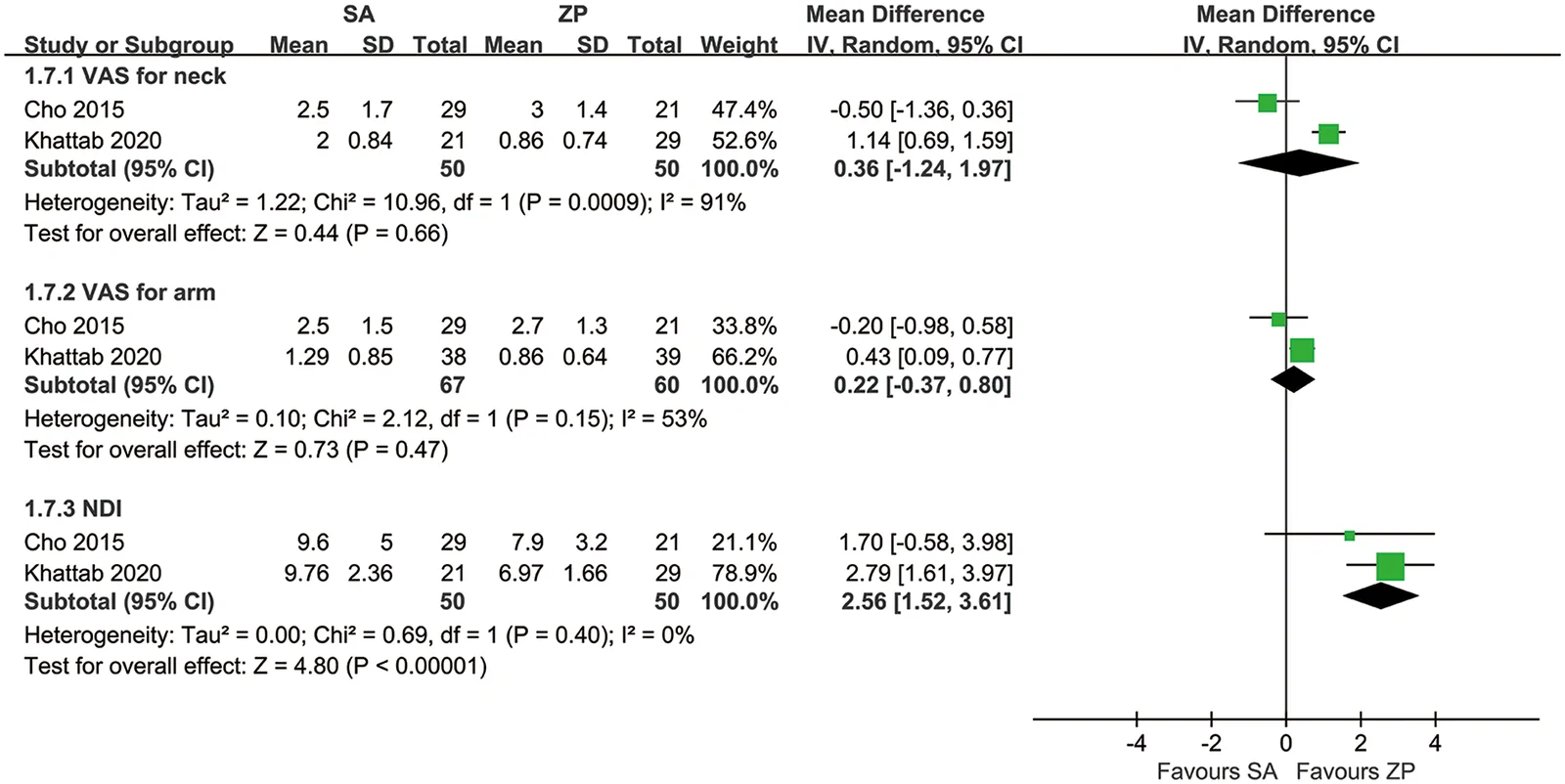
Comparison of clinical outcomes.

Pooled analysis of patient-reported outcomes revealed no statistically significant between-group differences in neck pain VAS score (MD: 0.36, 95% CI [−1.24, 1.97], *P* = 0.66, I^2^ = 91%, Figure 6) and arm pain VAS score (MD: 0.22, 95% CI [−0.37, 0.80], *P* = 0.47, I^2^ = 53%, Figure 6). The NDI score in SA was significantly higher than that in ZP group (MD: 2.56, 95% CI [1.52, 3.61], *P* < 0.00001, I^2^ = 0%, Figure 6).

#### 3.4.4 Fusion rate and subsidence rate

The criteria of subsidence and fusion was inconsistent. Regarding cage subsidence, different literatures adopted inconsistent cut-off thresholds: a reduction in intervertebral disc height of more than 2 mm or 3 mm relative to the immediate postoperative measurement was defined as subsidence in separate studies. In terms of bone fusion assessment, heterogeneous criteria were utilized among trials, including the Bridwell grading system, absence of prominent radiolucent zones surrounding the cage, presence of continuous trabecular bone bridging between vertebral endplates, and differences in interspinous motion measured on flexion-extension radiographs.

**Figure 7:**
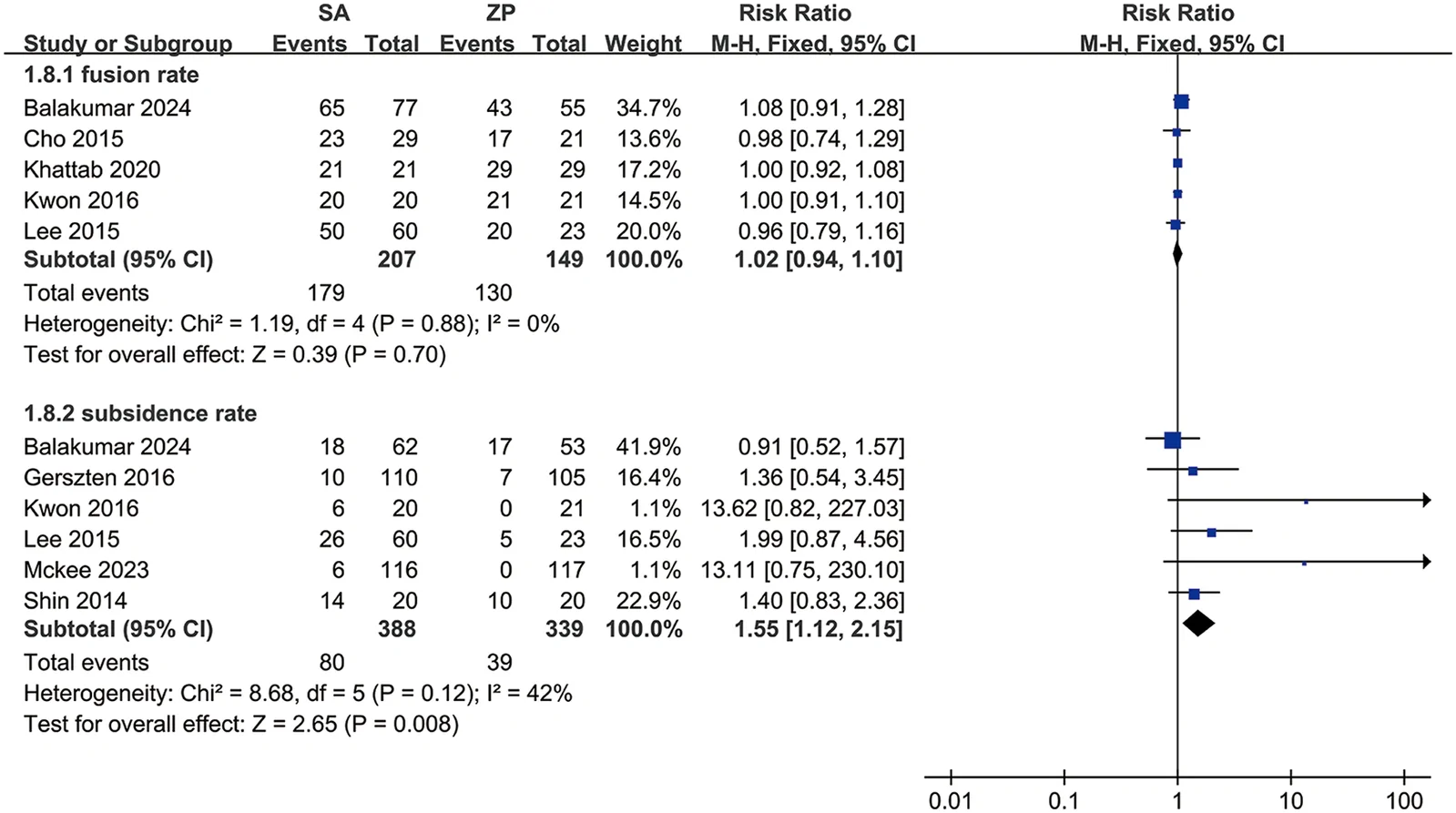
Comparison of the fusion rate and subsidence rate.

Pooled analysis revealed no significant difference in the fusion rate (RR: 1.02, 95% CI [0.94, 1.10], *P* = 0.70; I^2^ = 0%; Figure 7). In contrast, the rate of implant subsidence was significantly greater in SA than ZP (RR: 1.55, 95% CI [1.12, 2.15], *P* = 0.008; I^2^ = 42%; Figure 7).

**Figure 8:**
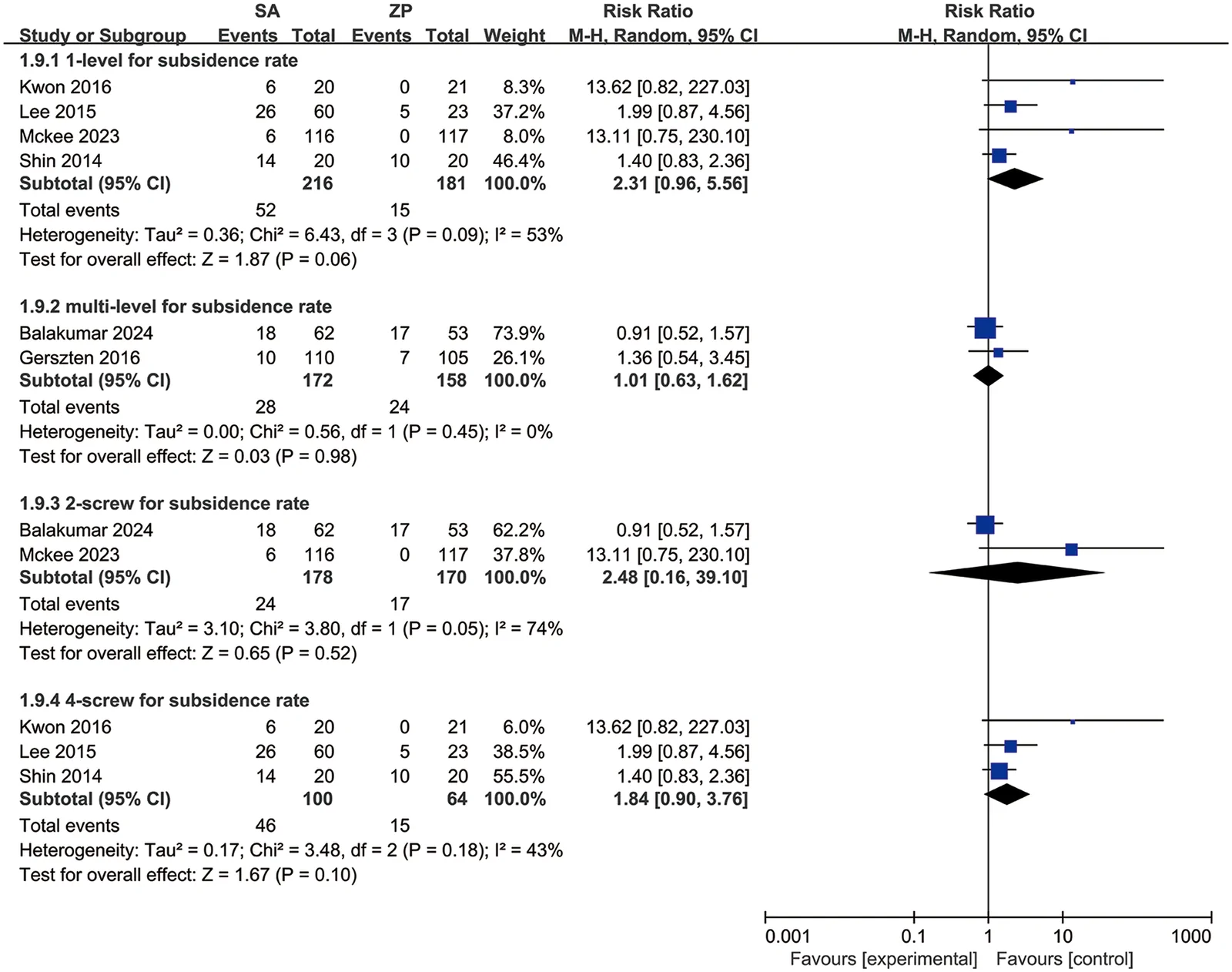
Subgroup analysis of subsidence rate according to number of surgical level and screws in ZP.

No statistical difference was identified in subsidence rate between the two groups in subgroup analysis of 1-level surgery (RR: 2.31, 95% CI [0.96, 5.56], *P* = 0.06; I^2^ = 53%; Figure 8), multilevel surgery (RR: 1.01, 95% CI [0.63, 1.62], *P* = 0.98; I^2^ = 0%; Figure 8), 2-screw ZP (RR: 2.48, 95% CI [0.16, 39.10], *P* = 0.52; I^2^ = 74%; Figure 8) and 4-screw ZP (RR: 1.84, 95% CI [0.90, 3.76], *P* = 0.10; I^2^ = 43%; Figure 8).

### 3.5 Publication bias assessment and meta-regression

Funnel plot-based evaluation of publication bias was not conducted, as fewer than 10 eligible studies were available for each individual outcome. Given that the number of included studies for each outcome variable was less than 10, meta-regression analysis was not performed.

### 3.6 Sensitivity analysis

For the outcome of the segmental Cobb angle, sensitivity analysis revealed that the original significant between-group difference was abolished after excluding the studies by Mckee et al. (35) and Shin et al. (36), indicating the unsatisfactory stability of this pooled result. In contrast, for all the other outcome comparisons, the pooled results remained unchanged with no significant fluctuations, even when each study was sequentially removed from the quantitative synthesis.

**Figure 9:**
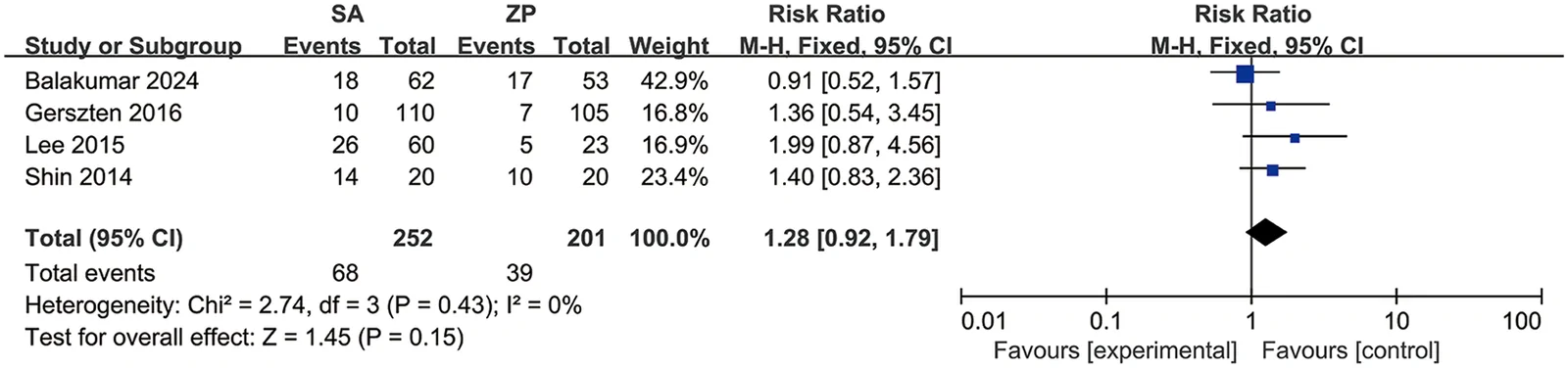
Sensitivity analysis by excluding zero-event studies for subsidence rate.

For the outcome of subsidence, sensitivity analysis was performed by excluding the two zero-event studies (33, 35). No statistical difference was identified in subsidence rate between the two groups (RR: 1.28, 95% CI [0.92, 1.79], *P* = 0.15; I^2^ = 0%; Figure 9).

### 3.7 Grade of evidence

The grade of the evidence of surgical time, intraoperative blood loss, NDI score, C2-7 Cobb angle, disc height, vertebral height, fusion rate and subsidence rate were considered low and the grade of the evidence of segmental Cobb angle and VAS score were considered very low (Table 2).

**Table 2:** GRADE certainty grading evaluation.

Outcomes	Certainty assessment							Certainty
	Number of Studies	Study design	Risk of bias	Inconsistency	Indirectness	Imprecision	Other	
Surgical time	2	NRS	not serious	not serious	not serious	not serious	none	⊕⊕◯◯ Low
Intraoperative blood loss	2	NRS	not serious	not serious	not serious	not serious	none	⊕⊕◯◯ Low
C2-7 Cobb angle	5	NRS	not serious	not serious	not serious	not serious	none	⊕⊕◯◯ Low
Segmental Cobb angle	4	NRS	not serious	high heterogeneity	not serious	not serious	none	⊕◯◯◯ Very low
Disc height	3	NRS	not serious	not serious	not serious	not serious	none	⊕⊕◯◯ Low
Vertebral height	2	NRS	not serious	not serious	not serious	not serious	none	⊕⊕◯◯ Low
VAS score	2	NRS	not serious	high heterogeneity	not serious	not serious	none	⊕◯◯◯ Very low
NDI score	2	NRS	not serious	not serious	not serious	not serious	none	⊕⊕◯◯ Low
Fusion rate	5	NRS	not serious	not serious	not serious	not serious	none	⊕⊕◯◯ low
Subsidence rate	6	NRS	not serious	not serious	not serious	not serious	none	⊕⊕◯◯ low

NRS, Non-randomized study.

## 4 Discussion

Few meta-analyses directly compare the safety and clinical and radiological efficacy of SA vs. ZP for the treatment of CDD. This study included a total of 2 studies reporting intraoperative blood loss and surgical time (30, 32). However, the pooled results of the meta-analysis in this study revealed that the surgical time of SA were significantly lower than those of ZP. Compared with the ZP, the SA does not require screw placement, with a shorter operation time, and surgical trauma is relatively minor. In contrast, both types of implants have significantly shorter operation times and less intraoperative blood loss than plates and cages do, and both are safe and minimally invasive surgical methods (37–39). The mean operative time differed by only 2.52 min between the SA and ZP groups. Although the differences reached statistical significance, they are of limited clinical relevance.

Only two studies reported the reoperation rate (29, 31). A total of 6 patients underwent reoperation, including 4 patients in the SA group and 2 patients in the ZP group. One patient underwent single-level surgery, and the remaining 5 patients underwent multilevel surgery. There was no statistically significant difference in the reoperation rate between the two groups. These findings indicate that both surgical methods are safe for the treatment of single-level cervical spondylosis. However, for patients who underwent multilevel surgery, reoperations occurred in both groups, and further research is needed regarding the selection of the appropriate surgical strategy.

There are few studies reporting comparisons of VAS and NDI scores between the two implants, with only two included, and they show significant heterogeneity and inconsistent results (30, 32). Cho et al. (30) retrospectively analyzed 50 patients with single-level cervical spondylosis treated with ACDF using ZP (21 patients) or SA (29 patients). They reported no statistically significant differences in neck VAS, arm VAS, or NDI scores between the two groups, and compared with the preoperative values, all the scores significantly improved. Khattab et al. (32) prospectively enrolled 50 patients with single-level cervical spondylosis treated with ACDF using ZP (29 patients) or SA (21 patients). The VAS and NDI scores were significantly greater in the SA group than in the ZP group at the 2-year follow-up. However, this study has certain limitations. The baseline characteristics of the included patients were uneven, and there were statistically significant differences in preoperative arm VAS and NDI scores between the two groups. This may account for the differences in postoperative outcomes and may also contribute to the high heterogeneity in this meta-analysis. Shin et al. (36) retrospectively compared the clinical efficacy of ACDF between the SA group and the ZP group on the basis of Odom's criteria, and no significant difference was detected. However, Lee et al. (34) reported different results. They evaluated clinical outcomes according to Odom's criteria and reported that the clinical efficacy of the SA group was significantly inferior to that of the Zero-P implant group (*P* = 0.049) and the plate and cage group (*P* = 0.026), whereas there was no statistically significant difference between the ZP group and the plate and cage group (*P* = 0.593). There are too few long-term follow-up studies on the improvement of clinical outcomes after SAS and ZP procedures, and further research is still needed.

The segmental Cobb angle, disc height and vertebral height in the SA group were significantly lower than those in the ZP group. The Zero-P implant was a stand-alone anchored cage with a plate and screw integrated into the intervertebral space. Thus, compared with the SA, the ZP was associated with better immediate stability and could better improve cervical alignment (34). Zhang et al. (40, 41) conducted a meta-analysis comparing the radiological efficacy between ZP and anterior plates with cages and reported that ZP and anterior plates with cages resulted in similar results in the maintenance of cervical lordosis in patients with one-level and two-level CDD. Scholz et al. (42) conducted biomechanical tests using C3-7 cadaveric specimens to compare the stability of SA and ZP in two- to three-level ACDF procedures. They reported that the Zero-P implant exhibited significantly better stability than the SA did in terms of flexion-extension, lateral bending, and rotational motion. No significant difference in the C2‒7 Cobb angle was detected between the two groups. However, the ZP tended to be superior to the SA in improving the overall cervical curvature. This may be because most of the included studies were single-level studies, so the overall impact on the cervical spine was minimal. Whether there is an effect on cervical alignment still needs to be confirmed by more high-quality studies.

No significant difference in the fusion rate was detected between the two groups. Achieving fusion is critical to successful ACDF surgery. Mu et al. (27) conducted a retrospective comparison of SA and ZP for two-level CDD, reporting no remarkable between-group difference in fusion rate (SA: 68/76; ZP: 71/78). Successful fusion may also play a crucial role in improving clinical outcomes (43). Elder et al. (44) retrospectively analyzed 22 patients with pseudarthrosis after ACDF and reported that all patients with pseudarthrosis presented with progressively worsening neck pain. After revision surgery, all patients achieved successful fusion, and compared with the preoperative values, the VAS scores significantly improved (*p* = 0.012).

The subsidence rate in the SA group was significantly greater than that in the ZP group. The immediate stability after SA implantation is inferior to that of the ZP group (42). Owing to the lack of plate and screw support, it is more prone to implant subsidence. The choice of implant used in ACDF surgery has a certain effect on postoperative cervical stability. Tome-Bermejo et al. (45) prospectively collected data from 56 patients who underwent ACCF surgery for cervical spondylosis. After a mean follow-up of 4.85 years, 83% of patients developed implant subsidence, including 7 patients with severe subsidence, which may adversely affect clinical outcomes. Milczynska et al. (46) retrospectively analyzed 39 patients with a total of 64 segments who underwent ACDF using stand-alone titanium cages. They reported that cage subsidence occurred in 9 patients and that there was no statistically significant difference in clinical functional scores between the subsidence group and the non-subsidence group. However, whether implant subsidence affects clinical symptoms remains highly controversial.

### 4.1 Limitations

Nevertheless, several nonnegligible limitations of the present meta-analysis warrant clarification. First, the overall sample size was relatively limited, with only 8 eligible studies enrolling a total of 727 patients included in the quantitative synthesis. All included studies were nonrandomized studies, leading to a restricted overall level of evidence. Second, the follow-up durations of included studies vary widely, ranging from only 3 months up to 24 months. Heterogeneous radiographic criteria are used to assess subsidence and fusion. The discrepancies in follow-up windows and outcome measurement criteria collectively amplify between-study heterogeneity; accordingly, all pooled radiological effect estimates should be interpreted with caution. Third, the included studies adopted inconsistent screw counts and surgical-level selections. Each subgroup analysis was derived from separate independent studies without formal tests for subgroup interaction. Significant confounding bias also exists between screw number and surgical level. Accordingly, this study cannot confirm that screw number or surgical level represents an independent influencing factor for treatment efficacy. The observed heterogeneity merely reflects baseline characteristics across studies and cannot support causal inference or effect-modifying conclusions. Thus, more high-quality randomized controlled trials are needed to further validate our findings.

## 5 Conclusion

Current pooled findings suggest that SA may be associated with shorter operative time, while ZP may correlate with lower subsidence rate and better preservation of cervical alignment. As all available evidence is derived exclusively from observational studies and rated as low to very low certainty, the conclusions must be interpreted with caution.

## Data Availability

The original contributions presented in the study are included in the article/Supplementary Material, further inquiries can be directed to the corresponding author/s.
