# Supplementary material for: Comparison of ACDF with stand-alone PEEK cage versus zero-p implant for the treatment of cervical degenerative disease: a systematic review and meta-analysis
Source: Front Surg. 2026 Sep 3;13:1945489. doi: 10.3389/fsurg.2026.1945489 (PMC13583156; doi:10.3389/fsurg.2026.1945489)
Supplement: Supplementary file 1 [file Table1.docx]

Supplementary Table 1 Search strategy

| Databases | Search No. | Index terms and Keywords |
| --- | --- | --- |
| PubMed | #1 | cervical [Title/Abstract] |
|  | #2 | standalone [Title/Abstract] OR stand alone [Title/Abstract] OR stand-alone [Title/Abstract] |
|  | #3 | stand-alone anchored spacer [Title/Abstract] OR anchored cage [Title/Abstract] OR self-lock* [Title/Abstract] OR SAAS [Title/Abstract] OR zero-p [Title/Abstract] OR zero-profile [Title/Abstract] OR no-profile [Title/Abstract] OR anchored spacer [Title/Abstract] |
|  | #4 | #1 AND #2 AND #3 |
| WOS | #1 | **TI=(**cervical**) OR AB=(**cervical**)** |
|  | #2 | **TI=(**standalone**) OR AB=(**standalone**) OR TI=(**stand alone**) OR AB=(**stand alone**) OR TI=(**stand-alone**) OR AB=(**stand-alone**)** |
|  | #3 | **TI=(**stand-alone anchored spacer**) OR AB=(**stand-alone anchored spacer**) OR TI=(**anchored cage**) OR AB=(**anchored cage**) OR TI=(**self-lock***) OR AB=(**self-lock***) OR TI=(SAAS) OR AB=(SAAS) OR TI=(**zero-p**) OR AB=(**zero-p**) OR TI=(**zero-profile**) OR AB=(**zero-profile**) OR TI=(**no-profile**) OR AB=(**no-profile**) OR TI=(**anchored spacer**) OR AB=(**anchored spacer**) OR** |
|  | #4 | #1 AND #2 AND #3 |
| EMBASE (via Ovid) | 1 | cervical.ab,ti. |
|  | 2 | standalone.ab,ti. |
|  | 3 | Stand alone.ab,ti. |
|  | 4 | Stand-alone.ab,ti. |
|  | 5 | stand-alone anchored spacer.ab,ti. |
|  | 6 | anchored cage.ab,ti. |
|  | 7 | self-lock*.ab,ti. |
|  | 8 | SAAS.ab,ti. |
|  | 9 | zero-p.ab,ti. |
|  | 10 | Zero-profile.ab,ti. |
|  | 11 | No-profile.ab,ti. |
|  | 12 | Anchored spacer.ab,ti. |
|  | 13 | 2 or 3 or 4 |
|  | 14 | 5 or 6 or 7 or 8 or 9 or 10 or 11 or 12 |
|  | 11 | 1 and 13 and 14 |
